# Pathobiological Implications of the Expression of EGFR, pAkt, NF-κB and MIC-1 in Prostate Cancer Stem Cells and Their Progenies

**DOI:** 10.1371/journal.pone.0031919

**Published:** 2012-02-23

**Authors:** Murielle Mimeault, Sonny L. Johansson, Surinder K. Batra

**Affiliations:** 1 Department of Biochemistry and Molecular Biology, College of Medicine, University of Nebraska Medical Center, Omaha, Nebraska, United States of America; 2 Department of Pathology and Microbiology, College of Medicine, University of Nebraska Medical Center, Omaha, Nebraska, United States of America; Wayne State University School of Medicine, United States of America

## Abstract

The progression of prostate cancers (PCs) to locally invasive, androgen-independent and metastatic disease states is generally associated with treatment resistance and disease relapse. The present study was undertaken to establish the possibility of using a combination of specific oncogenic products, including epidermal growth factor receptor (EGFR), pAkt, nuclear factor-kappaB (NF-κB) and macrophage inhibitory cytokine-1 (MIC-1) as biomarkers and therapeutic targets for optimizing the management of patients with localized PC at earlier disease stages. The immunohistochemical and immunofluorescence data have revealed that the expression levels of EGFR, Ser^473^-pAkt, NF-κB p65 and MIC-1 proteins were significantly enhanced in the same subset of 76 cases of prostatic adenocarcinoma specimens during the disease progression and these biomarkers were expressed in a small subpopulation of CD133^+^ PC cells and the bulk tumor mass of CD133^−^ PC cells. Importantly, all of these biomarkers were also overexpressed in 80–100% of 30 PC metastasis bone tissue specimens. Moreover, the results have indicated that the EGF-EGFR signaling pathway can provide critical functions for the self-renewal of side population (SP) cells endowed with stem cell-like features from highly invasive WPE1-NB26 cells. Of therapeutic interest, the targeting of EGFR, pAkt, NF-κB or MIC-1 was also effective at suppressing the basal and EGF-promoted prostasphere formation by SP WPE1-NB26 cells, inducing disintegration of SP cell-derived prostaspheres and decreasing the viability of SP and non-SP WPE1-NB26 cell fractions. Also, the targeting of these oncogenic products induced the caspase-dependent apoptosis in chemoresistant SP WPE1-NB26 cells and enhanced their sensibility to the cytotoxic effects induced by docetaxel. These findings suggest that the combined use of EGFR, pAkt, NF-κB and/or MIC-1 may represent promising strategies for improving the accuracy of current diagnostic and prognostic methods and efficacy of treatments of PC patients in considering the disease heterogeneity, thereby preventing PC progression to metastatic and lethal disease states.

## Introduction

Prostate cancer (PC) remains among the most frequently diagnosed solid tumors in men and the metastatic PC forms still represent the second leading cause of cancer-related death [1]–[5]. Important advances in past few years have led to an earlier diagnosis and effective therapeutic intervention by radical prostatectomy and/or radiation therapy for the patients with low-grade and organ-confined PCs [1], [4], [6], [7]. Disease progression to locally advanced, metastatic and castration-resistant prostate cancers (CRPCs) is associated with treatment resistance and disease relapse [1], [4], [6]. Although current anti-hormonal and chemotherapeutic regimens for highly invasive and metastatic PCs generally have improved the quality of life, these therapies are only palliative and culminate in the death of most patients after about 12–19 months following diagnosis [1], [4], [6].

Numerous studies have been performed to establish the etiopathological causes of PCs. The extrinsic and intrinsic factors pre-disposing to PC development include intense oxidative stress, inflammatory atrophies and fibrosis associated with severe tissue injuries, hormonal deregulation and more particularly with advancing age [8]–[13]. Initiation and progression of PC is generally characterized by a down-regulation of diverse tumor suppressor gene products, including phosphatase tensin homolog deleted on chromosome 10 (PTEN) and p53, combined with an up-regulation of the expression and/or activity of numerous oncogenic signaling elements in PC cells [4], [13], [14]. The interplay of complex signaling networks of distinct tumorigenic pathways initiated by hormones, growth factors, cytokines and chemokines through their cognate receptors is typically involved in the PC progression to locally advanced and metastatic disease [4], [10], [11], [13], [15]. Among the frequent deregulated gene products, the enhanced expression and activation of diverse receptor tyrosine kinases, including epidermal growth factor receptor (EGFR), during the epithelial-mesenchymal transition process may lead to the sustained activation of mitogen-activated protein kinases, phosphatidylinositol 3′-kinase (PI3K)/Akt, nuclear factor kappa-B (NF-κB) and macrophage inhibitory cytokine-1 (MIC-1) [4], [13], [14], [16]–[32]. These oncogenic products may cooperate to promote the sustained growth, survival, invasion and metastasis of PC cells as well as for their acquisition of androgen-independent (AI) and chemoresistant phenotypes, treatment resistance and disease recurrence [4], [13]–[16], [18]–[21], [23], [25]–[27], [30]–[39].

In addition, recent accumulating lines of experimental evidence have also revealed that PC stem/progenitor cells, also designated as PC- and metastasis-initiating cells, expressing stem cell-like markers such as CD133, CD44^high^, aldehyde dehydrogense “ALDH^high^” and/or CXC chemokine receptor 4 can provide critical functions in prostate carcinogenesis, metastases at distant sites and tumor re-growth and disease recurrence after treatment initiation [4], [10], [13], [14], [40]–[58]. It has been shown that highly tumorigenic PC stem/progenitor cells were able to give rise *in vitro* and *in vivo* to the bulk mass of differentiated PC cells expressing secretory luminal phenotypes, including androgen receptor and prostatic acid phosphatase, and reconstitute the tumors *in vivo* with a histological architecture of a Gleason grade comparable to the patient's original tumors [13], [40]–[45], [47], [48], [53]. It has also been observed that the PC stem/progenitor cells, including side population (SP) isolated from PC cells by using Hoechst dye efflux technique, which possess an AI phenotype and express high levels of ATP-binding cassette (ABC) multidrug transporters such as ABCG2, were also more resistant than their differentiated progenies and non-SP cells to the anti-hormonal and chemotherapeutic treatments [13], [14], [50]–[52], [59]. In spite of these advances, additional studies are required to validate distinct molecular biomarkers and therapeutic targets in PC stem/progenitor cells and their progenies that could be used in combination for optimizing the therapeutic management of PC patients at earlier disease stages.

The present investigation was undertaken to determine the clinical relevance of using a combination of multiple deregulated oncogenic products, including EGFR, the phosphorylated form of Akt, NF-*κ*B p65 and MIC-1 as molecular biomarkers to predict the risk of PC progression to locally advanced tumor and therapeutic targets to eradicate the total PC cell mass. Therefore, the immunohistochemical analyses of the expression levels and co-localization patterns of these proteins were made on the same panel of PC tissues and compared with non-malignant adjacent tissues and normal prostatic tissue specimens. Moreover, the prostasphere-forming and -disintegration assays and viability tests with SP cells endowed with stem cell-like properties and the non-SP cell fraction from the highly tumorigenic and invasive WPE1-NB26 cell line were performed with or without exogenous EGF in the absence or presence of the specific inhibitory agents of these oncogenic products. Overall, the results have supported the benefits of combining these oncogenic products as molecular biomarkers and therapeutic targets for improving the accuracy of diagnostic and prognostic methods and the efficacy of treatments of PC patients at earlier disease stages.

## Materials and Methods

### Materials

The human WPE1-NB26 cell line was originally obtained from American Type Culture Collection (Manassas, VA). The parental WPE1-NB26 cells were routinely maintained in keratinocyte serum-free medium (SFM) supplemented with antibiotics (100 UI/ml penicillin-100 µg/ml streptomycin), L-glutamine, bovine pituitary extract and epidermal growth factor (EGF) according to the manufacturer's instructions in a 37°C incubator supplied with 5% CO_2_. Keratinocyte-SFM, MitoTracker Red CMXRos dye and all other culture materials were from Life Technologies (Carlsbad, CA). Docetaxel, partenolide, Akt inhibitor VIII, and 3-(4,5-dimethylthiazol-2-yl)-2,5-diphenyltetrazolium bromide (MTT) were purchased from Sigma-Aldrich (St. Louis, MO), LY294002 from Calbiochem Corp (San Diego, CA) and gefitinib came from LC laboratory (Woburn, MA). The rabbit polyclonal anti-CD133 antibody (H-284), mouse monoclonal anti-CD44 (HCAM, F-4) antibody, rabbit polyclonal anti-ABCG2 antibody (B-25), rabbit polyclonal anti-EGFR antibody (1005), goat polyclonal anti-Tyr^1173^-phospho-EGFR antibody (1173) recognizing the EGFR form phosphorylated at tyrosine 1173 and rabbit polyclonal anti-NF-κB p65 protein subunit (C-20) were purchased from Santa Cruz Biotechnology, Inc (Santa Cruz, CA). The mouse monoclonal anti-β-actin antibody (clone AC-15) was provided by Sigma-Aldrich (St-Louis, MO, USA) and the rabbit monoclonal anti-Ser^473^-pAkt antibody (D9E) from Cell Signaling Technology, Inc. The rabbit polyclonal antibody directed against the cleaved caspase-9 fragment was purchased from Cell Signaling Technology (Danvers, MA, U.S.A.) and mouse monoclonal anti-cytochrome *c* (6H2) antibody provided by Santa Cruz Biotechnology, Inc (Santa Cruz, CA, U.S.A.). Rabbit polyclonal anti-MIC-1 antibody was generated in our laboratory against the C-terminal amino acid region of the mature MIC-1 protein as previously described [27], [29]. The phycoerythrin-conjugated monoclonal anti-CD133/2 antibody (293C3) was purchased from Miltenyi Biotec. Inc. and employed according to the manufacturer's instructions. The Vectastain avidin-biotin complex “ABC” method peroxidase kit and 3,3′-diaminobenzidine “DAB” substrate kit for the immunohistochemical staining were purchased from Vector Laboratories (Burlingame, CA).

### Immunohistochemical and double-immunohistofluorescence analyses

Immunohistochemical studies on the expression levels and cellular localization of EGFR, Ser^473^-pAkt, NF-κB p65 and MIC-1 proteins in non-malignant and malignant prostate tissues were carried out on prostate tissue microarrays made from formalin-fixed and paraffin-embedded tissues purchased from Biomax Inc. (Rockville, Maryland, USA) as previously described. The analyzed tissue specimens include the PR954 tissue microarray slide containing duplicate cores from 36 cases of patients with primary prostatic adenocarcinoma (Gleason scores: 6–10; stages T2–T4) and the corresponding matched non-malignant adjacent tissues from the same patients, and PR483 tissue microarray slide containing one core from 40 cases of patients with primary prostatic adenocarcinoma (Gleason scores: 6–10; stages T2–T4) and 8 normal prostate tissues from autopsy used as controls. In addition, the expression of all of biomarkers was also analyzed in 30 bone metastasis tissues from PC patients (Gleason scores: 6–10) (TriStar Technology Group, LLC, U.S.A.). The technique used for immunohistostaining has been previously described [36], [38], [52]. Briefly, the tissue sections were deparaffinized with EZ-DeWax™ (Bio Genex, San Ramon, CA) and rehydrated using graded ethanol solutions. After washing the slides 3 times with phosphate buffer saline (PBS) for 5 min, tissue sections were submerged in microwave antigen retrieval solution consisting of 0.01 M citrate buffer pH 6.0 and subjected to microwave irradiation 3 times during 3 min. The nonspecific immunostaining was blocked in diluted vectastain normal horse serum (Vector “ABC” kit) for 10 min and the slides were then incubated with primary anti-EGFR, -Ser^473^-pAkt, -NF-κB p65 protein subunit or -MIC-1 antibody in a humidified chamber overnight at 4°C. After washing with PBS, the slides were incubated with biotinylated universal secondary antibody for 30 min and rewashed with PBS. Endogenous peroxidase activity was quenched using 0.3% hydrogen peroxide in methanol∶PBS (1∶1) for 10 min. After an additional wash, the slides were incubated with ABC vectastain solution for 30 min. The tissue sections were submerged in a staining solution containing 3,3′-diaminobenzidine “DAB” substrate as indicated in the manufacturer's instructions and rinsed 3 times in water. A reddish-brown color precipitate observed on tissue sections indicates a positive immunoreactivity with the tested primary antibody. The slides were counterstained with hematoxylin, dehydrated and permanently mounted with vectamount permanent mounting media (Vector Laboratories). Images that were captured on a Nikon Eclipse E400 light microscope (Nikon Corporation, Tokyo, Japan) at different magnifications are representative of analyzed samples.

For each tissue section, the intensity of immunoreactivity for EGFR, Ser^473^-pAkt, NF-κB p65 or MIC-1 protein was semi-quantitatively graded by a urologic pathologist (Dr. Johansson) on a 0 to +3 scale (0 = no staining, 1+ = week staining, 2+ = moderate staining, and 3+ = strong staining). The percentage of PC cells positive for each biomarker analyzed within a given tissue core was also scored on a 1 to 4 scale (1 = 0–25% positive PC cells, 2 = 26–50% positive cells, 3 = 51–75% positive cells, and 4 = 76–100% positive cells). The score of the staining intensity and the percentage of immunoreactive PC cells were then multiplied to obtain a composite score ranging from 0 to 12. The staining intensity of different tested proteins in prostate adenocarcinoma samples was scored and compared to the normal prostate tissues, and the value was considered enhanced if the staining intensity was higher by one or more points.

In addition, the double-immunohistofluorescence analyses of the co-localization of stem cell-like marker, CD133 antigen (prominin-1) with unphosphorylated EGFR or its activated Tyr^1173^p-EGFR phosphorylated form, Ser^473^-pAkt, NF-κB p65 or MIC-1 were also carried out on deparaffinized and rehydrated non-malignant and malignant human prostatic tissue specimens from the patients obtained from UNMC's tissue bank as previously reported [52], [60]. The tissue slides were blocked in the presence of 10% goat serum for 30 min followed by incubation with the phycoerythrin-conjugated anti-CD133 antibody plus anti-EGFR, anti-Tyr^1173^-pEGFR, anti-Ser^473^-pAkt, anti-NF-*κ*B p65 or anti-MIC-1 antibody for 2 h. The slides were washed twice with PBS and processed for immunofluorescent detection as described below for the confocal microscopic analyses of fixed cells.

### Isolation of the SP and non-SP cell fractions and CD133^+^ PC cell subpopulation from human tumorigenic and invasive WPE1-NB26 cell line by flow cytometry

The parental WPE1-NB26 cells (1×10^6^ cells/mL) were stained with Hoechst buffer containing a final concentration of 2 µg/mL fluorescent Hoechst dye at 37°C for 2 h. The small subpopulations of SP and non-SP cells were isolated by fluorescence-activated cell sorting (FACS) as previously described [52], [60], [61]. The analyses and sorting of the viable SP and non-SP cell fractions were done using a FACS Aria flow cytometer with a DIVA software (Becton Dickinson Biosciences). The SP and non-SP cell fractions were collected after FACS and the expression level of the CD133 stem cell-like marker without apparent further phenotypic and differentiation changes in these two cultured cell subpopulations was obtained by maintaining the cells in serum-free keratinocyte culture medium containing exogenous EGF (10 ng/mL) before their use.

### Immunoblot analyses

The SP and non-SP cell lysates were prepared as previously described [36], [38], [60]. The protein concentrations were estimated by using a detergent-compatible protein assay kit from Bio-Rad Laboratories, Inc. (Hercules, CA). The samples corresponding to 20 µg proteins were resolved by electrophoresis on a 8 or 10% SDS-polyacrylamide gel under reducing conditions. The proteins were transferred onto an immobilon-P transfer membrane and blocked in 5% non-fat dry milk in PBS for 2 h and subjected to the standard immunodetection procedure. At the end of incubation, the blot was washed in TBST (50 mM Tris-HCl, pH 7.4, 150 mM NaCl and 0.05% Tween) and incubated with horseradish peroxidase-conjugated secondary antibody (Amersham Biosciences, Piscataway, NJ) for 1 h. Antibody-antigen complexes were visualized using enhanced chemiluminescence kit (Amersham Biosciences).

### Confocal microscopy analyses

The SP and non-SP cell fractions from the WPE1-NB26 cell line were grown at a low density on sterilized cover slips for 24 h, washed with PBS, and fixed in ice-cold methanol at −20°C for 2 min [36], [38], [52]. The cells were blocked in 10% goat serum for 30 min and incubated with phycoerythrin-conjugated monoclonal anti-CD133/2 antibody (293C3), mouse monoclonal anti-CD44 (HCAM, F-4) antibody, rabbit polyclonal anti-ABCG2 antibody (B-25), rabbit polyclonal anti-EGFR antibody (1105), goat polyclonal anti-Tyr^1173^-pEGFR antibody (1173), rabbit monoclonal anti-pAkt antibody (D9E), rabbit polyclonal anti-NF-κB antibody (C-20), rabbit polyclonal anti-MIC-1 antibody or mouse monoclonal anti-β-actin antibody (clone AC-15) diluted in PBS for 1 h at room temperature. After three washes with PBS, the cells were then incubated with fluorescein isothiocyanate (FITC)-conjugated goat anti-mouse, FITC-conjugated donkey anti-goat and/or Texas red-conjugated goat anti-rabbit secondary antibody (Jackson ImmunoResearch Laboratories, Inc., West Grove, PA) for 1 h. In addition, the SP cells treated with different cytotoxic agents during 4 days were stained with MitoTracker Red CMXRos in humidified chamber at 37°C in the dark for 30 min prior to the fixation and staining of SP cells with mouse monoclonal anti-cytochrome c antibody for 1 h followed by an incubation with FITC-conjugated goat anti-mouse for 1 h. Then, all of PC cells were washed again with PBS, nuclei counterstained with diamidino-2-phenylindole (DAPI) and mounted on glass slides in anti-fade Vestashield mounting medium (Vector Laboratories, Burlingame, CA). Immunofluorescence staining was observed under a confocal laser scanning microscope (LSM 410, Zeiss, Gottingen, Germany).

### Prostasphere-forming and disintegration assays

The SP and non-SP cell fractions isolated from the highly tumorigenic and invasive WPE1-NB26 cell mass were maintained in serum-free keratinocyte culture medium. The self-renewal capacity of SP cells *versus* the non-SP cell fraction isolated from the highly tumorigenic and invasive WPE1-NB26 cell line was estimated by their ability to form the non-adherent aggregates designated as prostaspheres in serum-free culture conditions under ultra-low attachment plate (Corning, invitrogen). For prostasphere-forming assays, 500 viable SP or non-SP cells obtained after cell sorting were suspended in serum free-keratinocyte medium without or with exogenous EGF (10 ng/ml) onto a 6-well ultra-low attachment plate in the absence or presence of different drugs. The tested drugs include a specific inhibitory agent of EGFR (gefitinib), PI3K (LY294002), pAkt (pAkt inhibitor VIII), NF-κB (partenolide) and MIC-1 (anti-MIC-1 antibody) as well as the current chemotherapeutic drug docetaxel. All samples were plated in triplicate. After 7 days of incubation, the numbers of prostaspheres formed were counted and the representative pictures of SP WPE1-NB26 cell-derived prostaspheres were photographed by using Accu-scope phase-contrast microscope at a magnification of 200×.

In addition, for the disintegration assays, 500 viable SP WPE1-NB26 cells were grown in serum-free culture conditions under an ultra-low attachment plate during 7 days for the formation of prostaspheres and then the tested drugs were added to culture medium and incubated for 4 additional days. At day 11, the representative pictures of disintegrated prostaspheres were photographed by using Accu-scope phase-contrast microscope at a magnification of 200×.

### TUNEL assay

The terminal deoxynucleotidedyl transferase dUTP nick end labeling (TUNEL) assay was performed to detect the DNA fragmentation indicative of apoptotic cell death induced by tested cytotoxic agents on the SP WPE1-NB26 cell subpopulation. After washing of fixed SP WPE1-NB26 cells with PBS, cells were incubated in the TdT reaction mixture consisting of nucleotide-labeling mix (TUNEL Label) contains fluorescein-dUTP and -dNTPs plus TdT enzyme (Roche diagnostics, IN) in humidified chamber at 37°C in the dark for 1 h. The SP cells were washed three times in PBS and incubated with a primary antibody directed against the cleaved capsase-9 fragment for 1 h at room temperature, followed by an incubation with FTIC-conjugate secondary antibody for 1 h. After three rinses with PBS, cells were counterstained with DAPI for nuclear stain and visualized by confocal fluorescence microscopy as above mentioned.

### Cell viability assays

For cell viability assays, the SP and non-SP cell fractions isolated from the WPE1-NB26 cell line were seeded on 96-well plates at a density of 3×10^4^ cells per well in a total volume of 200 µL free-serum keratinocyte culture medium as previously mentioned [36], [38], [52]. After 3 days, the SP and non-SP WPE1-NB26 cells were untreated or treated with different concentrations of tested drugs including gefitinib, LY294002, pAkt inhibitor VIII, partenolide, anti-MIC-1 antibody or docetaxel, alone or in combination. After 72 hours of incubation, the cell viability was estimated by a MTT colorimetric test.

### Flow cytofluorometric analyses

SP WPE1-NB26 cells were grown at a density of 5×10^5^ cells on 25 cm^2^ dishes as described previously. The SP cells were treated with different concentrations of tested drugs, alone or in combination with 5 nM docetaxel during four days. The apoptotic effect induced by tested agents on the SP WPE1-NB26 cells was estimated after DNA staining of each sample with the propidium iodide by FACS analyses as previously described [36], [38], [52].

### Statistical analyses

Statistical analyses were performed using the Student's *t*-test to compare the results with *P* values<0.05 indicating statistically significant differences. More specifically, immunohistochemical data were analyzed using Windows version 9.6.4.0. software. The composite scores of biomarker expression were considered as continous variables and compared using Student's two-tailed t test assuming unequal variance for independent samples.

## Results

### Immunohistochemical analyses of expression levels of EGFR, Ser^473^-pAkt, NF-κB p65 and MIC-1 signaling elements in non-malignant and malignant prostate tissue specimens

**Figure 1:**
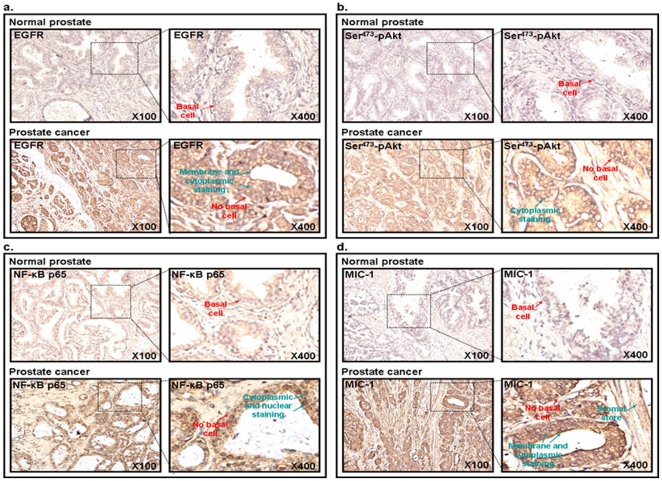
Immunohistochemical analyses of the expression levels of EGFR, Ser^473^-pAkt, NF-κB p65 and MIC-1 in non-malignant prostate and prostatic adenocarcinoma tissues. Microarray sections of non-malignant and malignant prostate tissue specimens were probed with an anti-EGFR, -Ser^473^-pAkt, -NF-κB p65 or -MIC-1 antibody after blocking with serum. All sections were examined under a microscope and the immunoreactivity was judged by dark brown staining. Representative pictures of stained tissue specimens of normal prostate, non-malignant adjacent tissues of prostatic tumor and prostatic adenocarcinoma obtained for (**a**) EGFR, (**b**) Ser^473^-pAkt, (**c**) NF-κB p65 and (**d**) MIC-1 are shown at original magnifications of ×100 and ×400. The arrows indicate the localization of basal cells in normal and non-malignant prostate epithelium and immunostaining detected for these biomarkers in prostatic adenocarcinoma tissue specimen. Moreover, the positive immunostaining detected for secreted MIC-1 protein in the stromal compartment adjacent to prostatic tumor tissue is also indicated.

**Figure 2:**
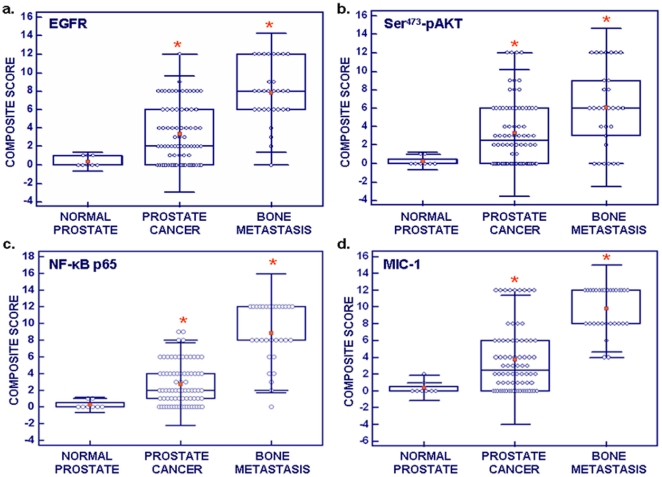
Comparison of the composite scores of expression levels of EGFR, Ser^473^-pAkt, NF-κB p65 and MIC-1 in non-malignant and malignant tissues from PC patients. Box plots showing the expression levels of (**a**) EGFR, (**b**) Ser^473^-pAkt, (**c**) NF-κB p65 and (**d**) MIC-1 during PC progression to metastatic disease stages. *, *P*<0.0001, indicates a significant increase between the composite scores obtained for prostatic adenocarcinoma and PC bone metastasis specimens relative to composite scores obtained for normal prostatic tissues.

The results from immunohistochemical analyses have indicated a very weak to undetectable immunostaining for EGFR, Ser^473^-pAkt, NF-κB p65 and MIC-1 in normal prostatic tissues of biopsy and adjacent non-malignant prostatic tissues from PC patients (Figure 1). In contrast, an enhanced expression of all of these biomarkers was detected in 66–75% of the 76 cases of prostatic adenocarcinomas (Gleason scores = 6–10) analyzed *versus* normal prostate tissues and associated with the stages (T2–T4) of the disease progression (Table 1). More particularly, a weak cytoplasmic and membrane immunostaining for the EGFR protein were detected only in a small number of basal and luminal prostatic epithelial cells in non-malignant prostatic tissues while its expression varied from moderate to strong within the cytoplasm and at the membrane respectively, in the malignant epithelial cells localized in the intermediate and luminal compartments in a subset of primary prostatic adenocarcinoma specimens (Figure 1a). The staining intensity associated with the EGFR protein expression was enhanced in 68% of 76 cases of primary prostatic adenocarcinoma specimens analyzed, as compared with the normal prostatic tissue from biopsy (Table 1). Moreover, the composite score value obtained for EGFR expression in PC specimens (3.4±0.4) was significantly superior to the value for normal prostate tissues (0.4±0.2); *p<0.0001) (Figure 2a). As shown in Figure 1b and c, the activated Ser^473^-pAkt phosphorylated form was also overexpressed in 66% of 76 cases of the prostatic adenocarcinomas analyzed and detected in the cytoplasm in PC epithelial cells whereas an enhanced expression of the p65 subunit NF-κB transcription factor occurred in 75% of 76 cases of prostatic adenocarcinomas and was mainly detected in cytoplasm and nuclei of PC epithelial cells. The composite score values obtained for Ser^473^-pAkt and NF-kB p65 expression in PC specimens (3.3±0.4 and 2.7±0.3) were significantly enhanced as compared to the value for normal prostate tissues (0.3±0.1 and 0.3±0.2; p<0.0001), respectively (Figure 2b and c). In addition, a stronger positive immunostaining was also seen for the MIC-1 protein in the cytoplasm and near the membrane in PC epithelial cells as well as for secreted MIC-1 in tumor stroma in 71% of 76 cases of prostatic adenocarcinomas as compared to normal and adjacent non-malignant prostate tissues analyzed (Figure 1d). The composite score value obtained for MIC-1 expression in PC specimens (3.7±0.4.) was significantly enhanced relative to the value for normal prostate tissues (0.4±0.3; *p<0.0001) (Figure 2d). Importantly, the results have also indicated that Ser^473^-pAkt, NF-κB p65 and MIC-1 were co-expressed with EGFR in the same subset corresponding at about 54–62% of PC tissue specimens analyzed suggesting that these oncogenic signaling elements may all cooperate to promote the malignant transformation of PC epithelial cells during disease progression to a locally advanced disease state (Table 1).

**Table 1:** Immunohistochemical analyses of biomarker expression levels in prostate adenocarcinoma tissues and PC bone metastasis specimens relative to normal prostate tissues.

Pathological diagnosis	aNumber of cases	Positive staining for EGFR	Positive staining for Ser^473^-pAkt	Positive staining for NF-κB p65	Positive staining for MIC-1
Adenocarcinoma	T2/42 cases	55%	62%	69%	71%
Adenocarcinoma	T3/30 cases	83%	80%	80%	68%
Adenocarcinoma	T4/4 cases	100%	100%	100%	100%
Adenocarcinoma	T2-T4/Total: 76 cases	68%	66%	75%	71%
Adenocarcinoma	**T2-T4/Total: 76 cases**	**—**	**54% co-expressed with EGFR**	**62% co-expressed with EGFR**	**57% co-expressed with EGFR**
PC bone metastasis	Total: 30 cases	93%	80%	93%	100%

a **T-primary tumor:** T2, tumor confined within prostate; T3, tumor extends through the prostate caspsule and T4, Tumor is fixed or invades adjacent structures other than seminal vesicles.

**Figure 3:**
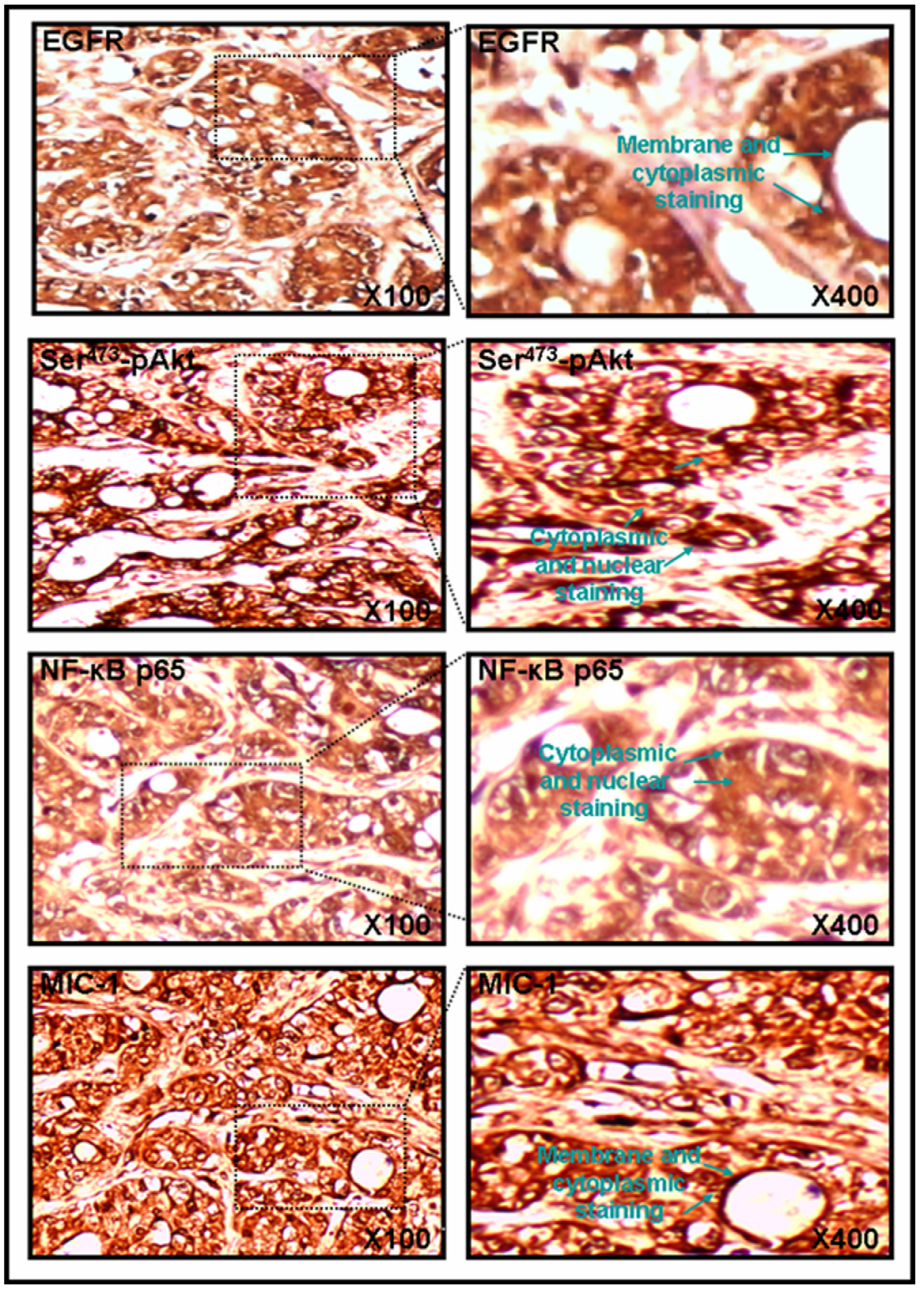
Immunohistochemical analyses of the expression levels of EGFR, Ser^473^-pAkt, NF-κB p65 and MIC-1 in PC bone metastasis tissues. Microarray sections of PC bone metastasis tissue specimens were probed with anti-EGFR, -Ser^473^-pAkt, -NF-κB p65 or -MIC-1 antibody after blocking with serum. All sections were examined under a microscope and the immunoreactivity was judged by dark brown staining. Representative pictures of stained PC bone tissue specimens obtained for EGFR, Ser^473^-pAkt, NF-κB p65 or MIC-1 are shown at original magnifications of ×100 and ×400.

Importantly, a positive immunostaining varying from moderate to strong, within the cytoplasm and near the membrane or in nuclei was also detected for EGFR, Ser^473^-pAkt, NF-κB p65 and MIC-1 in PC cells in 80–100% of bone metastasis tissues analyzed from 30 PC patients (Gleason scores = 6–10) (Figure 3; Table 1). Moreover, MIC-1 immunostaining varied from very weak to moderate intensity was seen in the stroma of PC bone metastasis tissues (Figure 3d). The composite scores obtained for the expression of all of these biomarkers in bone metastasis tissues from PC patients were also superior to the values observed for normal prostatic tissues and prostatic adenocarcinoma speciments (Figure 2; *p<0.0001).

### Double immunohistofluorescence confocal microscopy analyses of the expression level of the CD133 stem cell-like marker and its co-localization with EGFR, Ser^473^-pAkt, NF-κB p65 and MIC-1 signaling elements in non-malignant and malignant prostate tissue specimens

**Figure 4:**
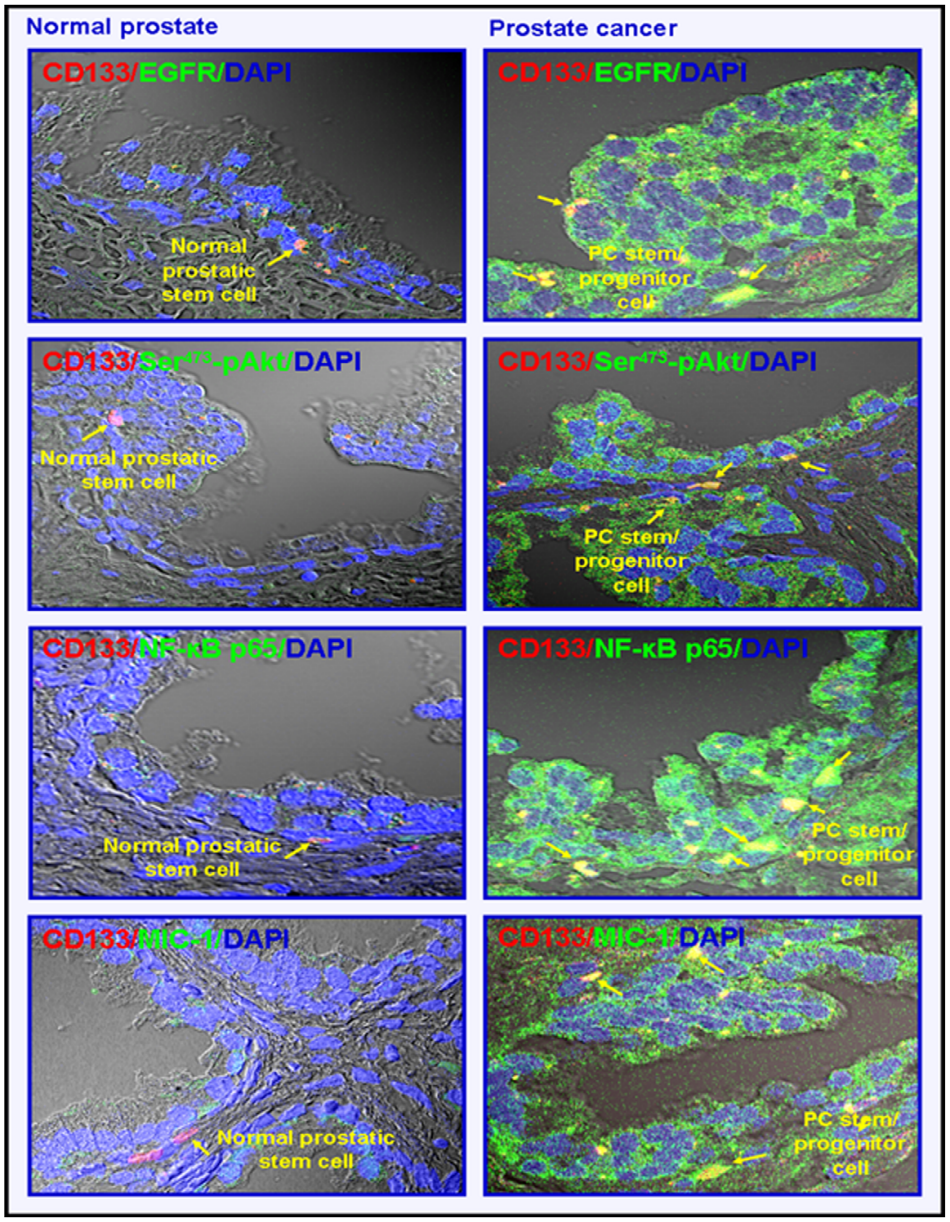
Immunofluorescence analyses of expression levels of EGFR, Ser^473^-pAkt, NF-κB p65 and MIC-1 signaling elements and their co-localization with a CD133 stem cell-like marker in non-malignant and malignant prostatic tissues. The double immunofluorescence analyses of the co-localization of the expression of markers in normal prostate and prostatic adenocarcinoma specimens from patients was simultaneously done with fluorescein-labeled anti-EGFR, -Ser^473^-pAkt, -NF-κB p65 or -MIC-1 antibody (green) plus phycoerythrin-labeled anti-CD133 antibody (red) after blocking with goat serum as described in Materials and Methods. The arrows indicate a double staining (yellow/purple) detected by confocal analyses, which is indicative of the co-localization of these markers. Representative pictures are shown at the original magnification of ×630.

To obtain experimental evidence of the potential implication of the enhanced expression and/or activation of EGFR, pAkt, NF-κB p65 and MIC-1 in the malignant transformation of CD133^+^ adult prostatic stem/progenitor cells into CD133^+^ PC stem/progenitor cells, we have also characterized the co-localization of the CD133 stem cell-like marker with these oncogenic signaling elements in the non-malignant and malignant prostatic tissues from patients (Figure 4). The results from double immunohistofluorescence analyses have revealed that the expression levels of all of these biomarkers were significantly enhanced in a small subset of CD133^+^ PC cells dispersed through the intermediate compartment in malignant prostate tissues as well as the bulk tumor mass of CD133^−^ PC cells in prostatic adenocarcinomas relative to non-malignant prostate tissue specimens from patients (Figure 4 and Figure S1). More specifically, a positive immunoreactivity was observed for EGFR and its phosphorylated Tyr^1173^-pEGFR activated form, Ser^473^-pAkt and MIC-1 in the cytoplasm and near or at the cell surface in intermediate and luminal PC cells detected in prostatic adenocarcinoma specimens (Figure 4 and Figure S1). Furthermore, a positive cytoplasmic and nuclear staining was also detected in intermediate and luminal PC cells for the NF-κB p65 subunit, which acts as a transcriptional signaling effector of numerous growth factor cascades including the activated EGFR pathway (Figure 4).

### Western blot and immunofluorescence analyses of expression levels of stem cell-like markers and EGFR, Ser^473^-pAkt, NF-κB p65 and MIC-1 in SP and non-SP cell fractions from parental WPE1-NB26 cell line

**Figure 5:**
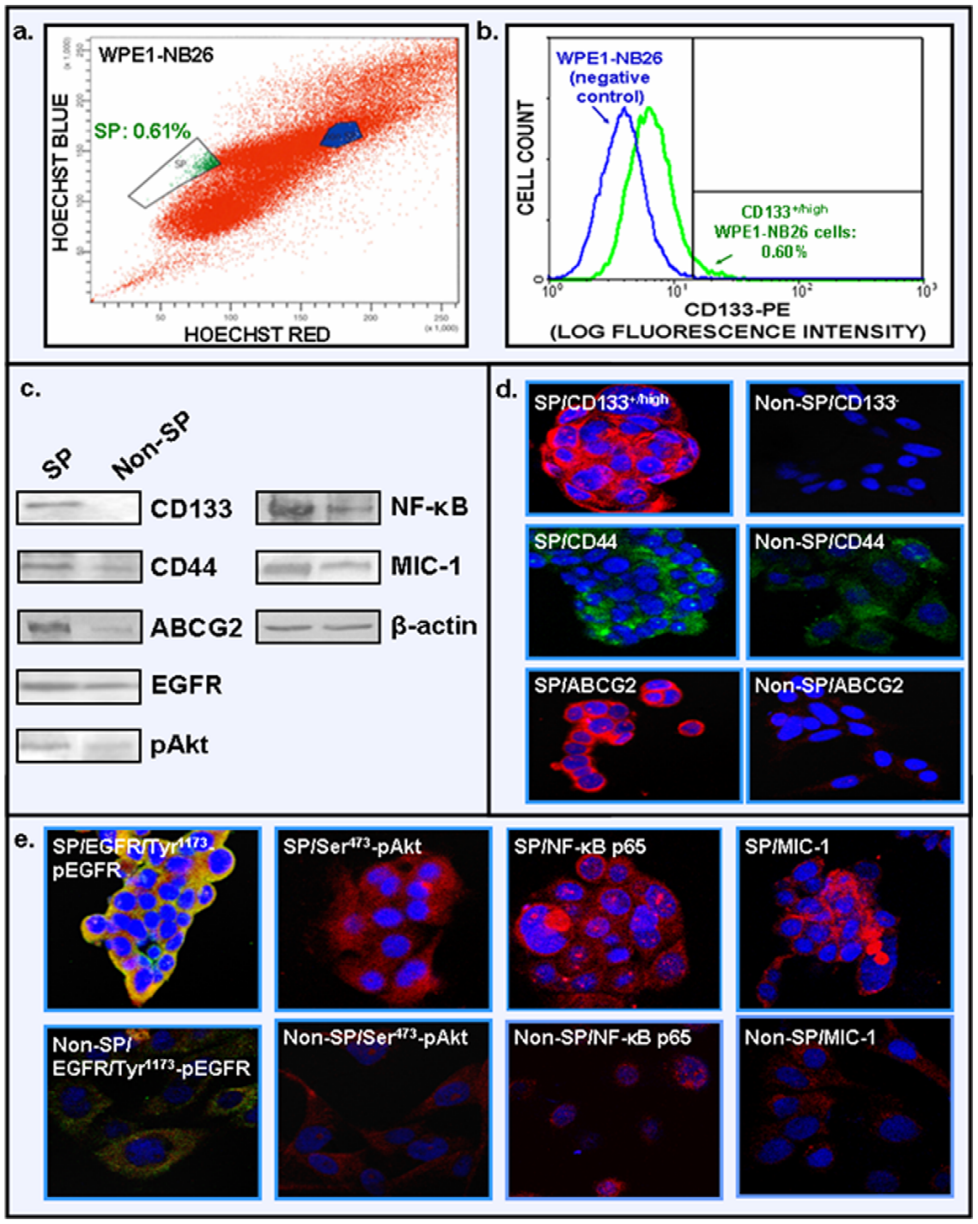
Characterization of phenotypic features of SP and non-SP cell fractions from parental tumorigenic and invasive WPE1-NB26 cells by the Hoechst dye efflux technique and FACS. **a**) Representative data of the Hoechst dye efflux profile obtained after staining of parental WPE1-NB26 cell line with fluorescent Hoechst dye showing the SP cell subpopulation (green) and non-SP fraction (blue) detected in the total mass of parental WPE1-NB26 cells. **b**) FACS profiles obtained after staining of parental WPE1-NB26 cells with phycoerythrin -labeled anti-CD133 antibody showing the percentage of CD133^+^ and CD133^−^ PC cells detected in the total mass of parental WPE1-NB26 cells. **c**) Comparative Western blot analyses of expression levels of prostatic stem cell-like markers (CD133 and CD44), multidrug transporter ABCG2, EGFR, Ser^473^-pAkt, NF-κB p65 subunit and secreted MIC-1 proteins detected in the SP and non-SP cell fractions isolated from parental WPE1-NB26 cell line. **d**) Immunofluorescence staining of methanol-fixed prostaspheres derived from SP cells and the adherent non-SP cell fraction isolated from the parental WPE1-NB26 cell line were done with anti-EGFR plus Tyr^1173^-pEGFR, Ser^473^-pAkt, NF-κB p65 or MIC-1 primary antibody plus fluorescein (green) and/or Texas red secondary antibody (red) and 4′,6-diamidino-2-phenylindole (nuclear blue) after blocking with goat serum. Representative pictures showing the expression level and cellular localization obtained for the stem cell-like markers including CD133 (red), CD44 (green) and ABCG2 (red) as well as overlaps of EGFR/Tyr^1173^-pEGFR (red/green, hybrid yellow), Ser^473^-pAkt (red), NF-κB p65 (red) and MIC-1 (red) are shown at the original magnification of ×630.

To further assess whether an enhanced expression and/or activation of EGFR, PI3K/Akt, NF-κB and MIC-1 occur in PC stem/progenitor cells during disease progression, a characterization of the expression levels of these signaling components was performed using SP and non-SP cell fractions from highly tumorigenic and invasive WPE1-NB26 cell line. The characterization of the parental WPE1-NB26 cell line by Hoechst dye efflux technique has revealed the presence of a SP subpopulation with a high Hoechst dye exclusion capacity corresponding to about 0.61% of total WPE1-NB26 cell mass (Figure 5a). Moreover, the FACS analyses after staining of WPE1-NB26 cells with phycoerythrin-labeled CD133 antibody has also indicated the presence of a small WPE1-NB26 cell population expressing a high level of CD133 stem cell-like marker corresponding to about 0.60% of the total PC cell mass (Figure 5b). The characterization of phenotypic features of the SP cell subpopulation from parental WPE1-NB26 cells by Western blot and immunofluorescence analyses has also revealed that these immature cells expressed higher levels of CD133 and CD44 stem cell-like markers and ABC multidrug transporter ABCG2 relative to the non-SP WPE1-NB26 cell fraction (Figure 5c and d). Importantly, the SP and non-SP WPE1-NB26 cell fractions also expressed different drug resistance-associated molecules, including EGFR, Ser^473^-pAkt, NF-κB p65 and MIC-1 (Figure 5c and d).

### Determination of the functions of EGFR, PI3K/pAkt, NF-κB and MIC-1 signaling elements for the self-renewal of SP cells *versus* the non-SP cell fraction from WPE1-NB26 cell line

**Figure 6:**
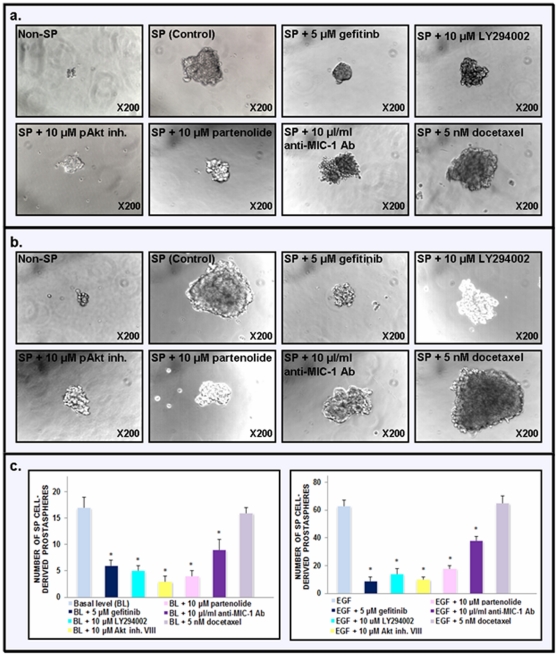
Determination of the effects induced by different drugs on the prostasphere-forming ability of the SP cell fraction from highly invasive and tumorigenic WPE1-NB26 cell line. The SP and non-SP cell fractions from the WPE1-NB26 cell line were subjected to the prostasphere formation culture on an ultra-low attachment plate in serum-free keratinocyte medium. The representative pictures of the dense prostaspheres formed by SP WPE1-NB26 cells (**a**) without or (**b**) after a treatment with exogenous EGF as compared to diffuse, abortive and very small aggregates formed by non-SP WPE1-NB26 cells are shown at a similar magnification of ×200. Moreover, the representative pictures of the prostaspheres formed by the SP WPE1-NB26 cell fraction (**a**) without or (**b**) after a treatment with exogenous EGF in the presence of different drugs, including a specific inhibitory agent of EGFR (gefitinib), PI3K (LY294002), pAkt (pAkt inhibitor VIII), NF-κB (partenolide), MIC-1 (anti-MIC-1 antibody) or docetaxel, are also shown at a similar magnification of ×200. The quantitative data of the number of prostaspheres formed by the SP WPE1-NB26 cell fraction (**c**) without or (**d**) after a treatment with exogenous EGF in the absence (control) or presence of different inhibitory agents obtained from at least 3 separate experiments are shown.

The establishment of the functions of EGFR, PI3K/pAkt, NF-κB and MIC-1 for the self-renewal of the SP cell subpopulation *versus* the non-SP cell fraction isolated from the highly tumorigenic and invasive WPE1-NB26 cell line was done by performing prostasphere-forming assays in the absence or presence of specific inhibitory agents of these signaling elements in serum free-keratinocyte medium under an ultra-low attachment plate. As shown in Figure 6a, the results from prostasphere-forming assays have revealed that the SP cells from the WPE1-NB26 cell line were able to generate many dense prostaspheres with a large size in culture after 7 days in the absence of EGF. Moreover, the addition of exogenous EGF into culture medium significantly promoted the number and size of prostaspheres formed by SP WPE1-NB26 cells indicating an important role of EGF-EGFR system for the self-renewal of these immature PC cells (Figure 6b). In contrast, the non-SP WPE1-NB26 cell fraction after FAC sorting formed only a small number of diffuse, abortive and very small primary prostaspheres in the absence or presence of EGF, while no secondary prostasphere was formed at the second passage as compared to the high prostasphere-forming capacity of SP WPE1-NB26 cells that was retained upon serial passage (Figure 6a and b). We have also observed that the 10 µl/ml of the pre-immune rabbit serum was used as control has not significant effect on the prostasphere-forming ability of SP WPE1-NB26 cells as compared to 10 µl/ml anti-MIC-1 antibody (data not shown). Also, the treatment with docetaxel has not significant effect on the prostasphere-forming ability of SP WPE1-NB26 cells in the absence or presence of EGF (Figure 6a and b).

Importantly, the data have also indicated that the number and size of the primary prostaspheres formed by CD133^+^ SP WPE1-NB26 cells without (basal level) and with EGF were significantly reduced in the presence of different concentrations of specific inhibitory agents of EGFR (gefitinib), PI3K (LY294002), pAkt (Akt inhibitor VIII), NF-κB (partenolide) or MIC-1 (anti-MIC-1 antibody) (Figure 6a, b and c). In particular, the prostasphere numbers were reduced up at 70% in the presence of 5 µM gefitinib, 10 µM LY294002, 10 µM Akt inhibitor VIII or 10 µM partenolide and about 60% for 10 µl/ml of the anti-MIC-1 antibody, indicating that EGF/EGFR, PI3K/Akt, NF-κB and MIC-1 signaling elements can contribute to the high self-renewal capacity of SP WPE1-NB26 cells (Figure 6c).

### Determination of the cytotoxic effects induced through the inhibition of EGFR, PI3K/pAkt, NF-κB and MIC-1 signaling elements on SP cells *versus* the non-SP cell fraction from WPE1-NB26 cell line

**Figure 7:**
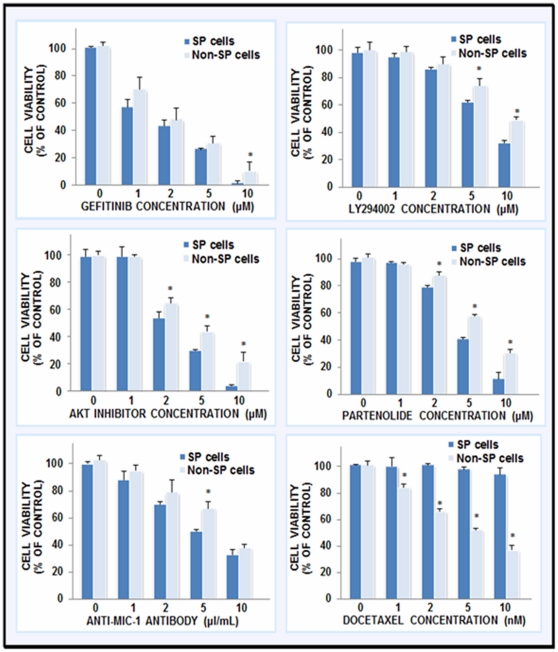
Cytotoxic effects induced by different tested drugs on SP and non-SP cell fractions isolated from highly tumorigenic and invasive WPE1-NB26 cells. The SP and non-SP WPE1-NB26 cell fractions were untreated or treated with the indicated concentrations of gefitinib, LY294002, pAkt inhibitor VIII, partenolide, anti-MIC-1 antibody or docetaxel for 72 hours and the cell viability was analyzed by MTT tests. The data are the means of at least three different experiments done in triplicate. *, *P*<0.05, indicates a significant difference between the cytotoxic effects induced by individual drugs on the SP cells *versus* the non-SP WPE1-NB26 cell fraction.

**Figure 8:**
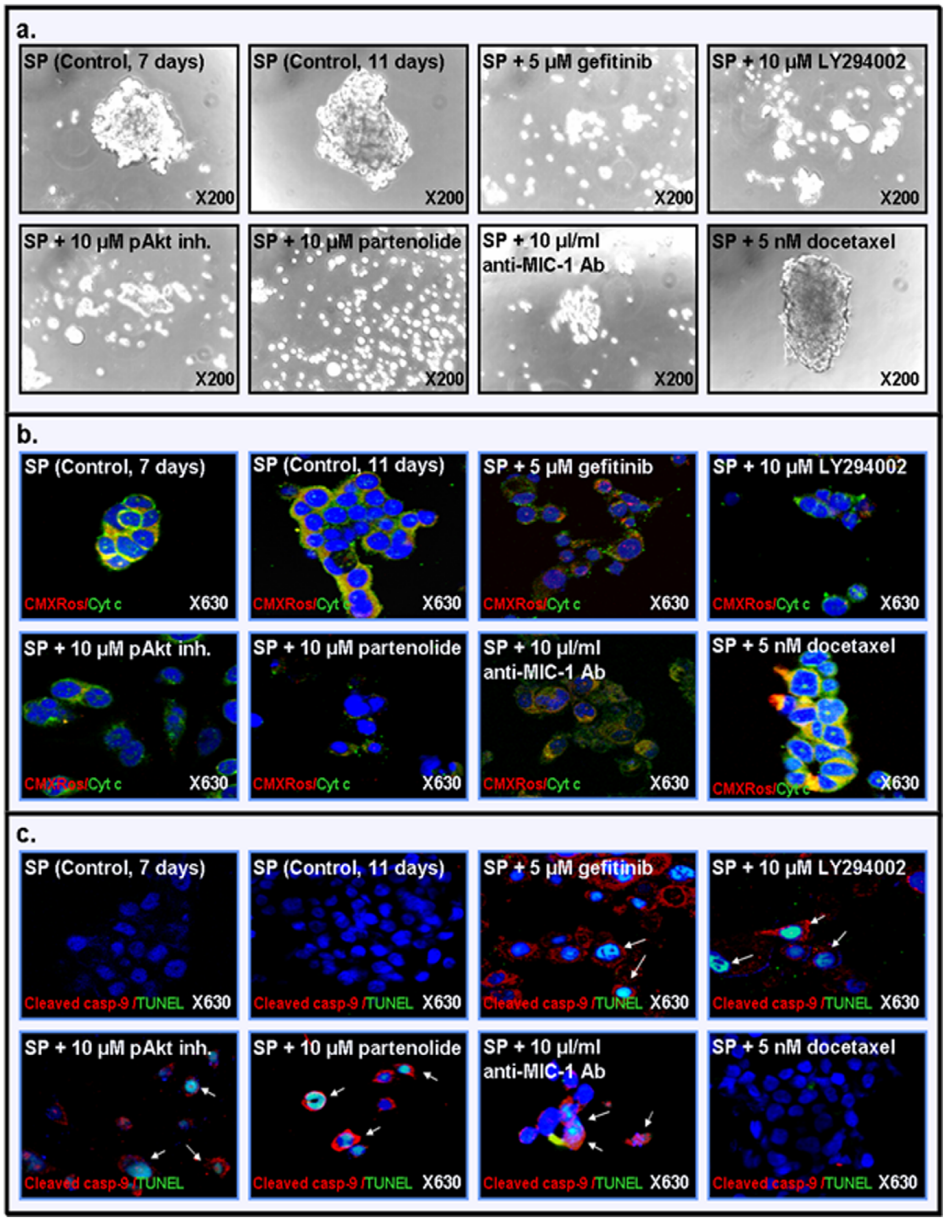
Estimation of the disintegration effects induced by different tested drugs on prostaspheres derived from SP WPE1-NB26 cell-and implication of the mitochondrial and caspase pathways. **a**) Representative pictures of the disintegration effect induced by a treatment with tested drugs during 4 days on the prostaspheres derived from SP WPE1-NB26 cells. **b**) Immunofluorescence staining of SP WPE1-NB26 cells after a treatment with indicated cytotoxic agents was done with anti-cytochrome c primary antibody plus fluorescein (green) secondary antibody and the mitochondria and nuclei stained with MitoTracker Red CMXRos (red) and DAPI (blue), respectively. Representative pictures showing the expression level and cellular localization of mitochondria (red), cytochrome c in mitochondria (green/red; hybrid yellow) or cytoplasm (diffuse green staining) are shown at the original magnification of ×630. **c**) Immunofluorescence staining of SP WPE1-NB26 cells after a treatment with indicated cytotoxic agents was done with a primary antibody directed against the cleaved caspase-9 fragment plus Texas red secondary antibody and TUNEL reactive mixture (green) and the nuclei counterstained with DAPI (blue). Representative pictures showing the expression level and cellular localization of the cleaved caspase-9 fragment (red) and TUNEL and DAPI (green/blue; hybrid cyan) in SP WPE1-NB26 cells are shown at original magnification ×630. The overlaps of double nuclear staining with TUNEL and DAPI (green/blue; hybrid cyan) associated with DNA fragmentation which is indicative of the apoptotic nuclei in SP cells are indicated by arrows. **d**) The SP WPE1-NB26 cells were untreated or treated with the indicated concentrations of a specific inhibitory agent including EGFR (gefitinib), PI3K (LY294002), pAkt (pAkt inhibitor VIII), NF-κB (partenolide) and MIC-1 (anti-MIC-1 antibody), alone or in combination with 5 nM docetaxel for 4 days, and the apoptotic cell death was analyzed by FACS. The panel shows the apoptotic effect induced by the tested agents that are expressed as the percentage of apoptotic SP WPE1-NB26 cells compared to non-treated SP cells (control). *, *P*<0.05, indicates a significant difference between the apoptotic effect induced by tested drugs plus 5 nM docetaxel *versus* individual drugs on the SP WPE1-NB26 cell fraction.

The results from MTT analyses have indicated that a treatment with increasing concentrations of gefitinib, Akt inhibitor VIII, partenolide or anti-MIC-1 antibody for 72 hours significantly reduced the number of viable SP and non-SP WPE1-NB26 cells (Figure 7). We have also observed that 10 µl/ml of the pre-immune rabbit serum used as control has not significant effect on the viability of cells as compared to 10 µl/ml anti-MIC-1 antibody (data not shown). Moreover, a treatment with the current chemotherapeutic drug docetaxel induced only a small cytotoxic effect on the SP WPE1-NB26 cell fraction while it markedly reduced the number of viable non-SP WPE1-NB26 cells (Figure 7). As shown in Figure 8a, a treatment with gefitinib, LY294002, Akt inhibitor VIII, partenolide or anti-MIC-1 antibody for 4 days was also effective at inducing the disintegration of the dense prostaspheres generated by SP WPE1-NB26 cells after 7 days of cultures under ultra-low attachment plate while docetaxel had no significant effect (Figure 8a).

In addition, in order to further investigate whether the suppression of EGFR, PI3K/pAkt, NF-κB, MIC-1 activity with specific inhibitory agents induced the cytotoxic effects on SP cells *via* a mitochondria- and caspase-dependent apoptotic pathway, the double-staining of cytochrome c plus MitoTracker Red CMXRos dye that specifically stains the mitochondria as well as the cleaved caspase-9 fragment plus TUNEL were performed. The data of double-immunofluorescence analyses of cellular localization of cytochrome c and MitoTracker Red have indicated that the cytotoxic effects induced by tested agents on SP WPE1-NB26 cells were associated with a loss of mitochondrial transmembrane potential (ΔΨm depolarization) as visualized by a decrease of the red staining for membrane potential sensitive dye, CMXRos in treated SP cells as compared to untreated SP cells used as controls (Figure 8b). The SP cells treated with tested cytotoxic agents also showed a diffuse green staining for cytochome c indicative of the release of cytochrome c from mitochondria into cytoplasm as compared to untreated SP cells (Figure 8b). Furthermore, the results of double-staining for the cleaved caspase-9 fragment and TUNEL have also revealed that the cytotoxic effects induced by tested agents was accompanied by an increase of the number of cleaved caspase-9 fragment- and TUNEL-positive SP WPE1-NB26 cells (Figure 8c). Together these date suggest that the tested cytotoxic agents may induce the cytochrome c release from mitochondria, caspase activation and DNA fragmentation in SP WPE1-NB26 cells which may results into the apoptotic death of some treated SP cells.

**Figure 9:**
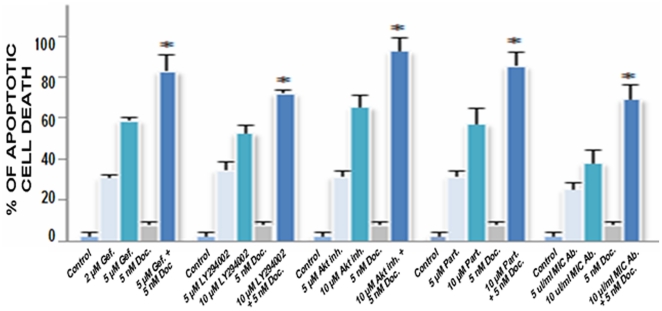
Quantitative analyses of apoptotic effects induced by different tested drugs, alone or in combination with docetaxel, on SP cells isolated from highly tumorigenic and invasive WPE1-NB26 cells. The SP WPE1-NB26 cells were untreated or treated with the indicated concentrations of a specific inhibitory agent including EGFR (gefitinib), PI3K (LY294002), pAkt (pAkt inhibitor VIII), NF-κB (partenolide) and MIC-1 (anti-MIC-1 antibody), alone or in combination with 5 nM docetaxel for 4 days, and the apoptotic cell death was analyzed by FACS. The panel shows the apoptotic effect induced by the tested agents that are expressed as the percentage of apoptotic SP WPE1-NB26 cells compared to non-treated SP cells (control). *, *P*<0.05, indicates a significant difference between the apoptotic effect induced by tested drugs plus 5 nM docetaxel *versus* individual drugs on the SP WPE1-NB26 cell fraction.

In support with this, the quantitative analyses of the number of cells detected in the sub-G1 phase by FACS have indicated that increasing concentrations of the tested drugs induced a high rate of apoptotic death on SP WPE1-NB26 cells as compared to untreated SP WPE1-NB26 used as control (Figure 9). Importantly, a combination of 5 µM gefitinib, 10 µM LY294002, 10 µM Akt inhibitor VIII, 10 µM partenolide or 10 µl/mL of anti-MIC-1 antibody with 5 nM docetaxel was also more effective than individual drugs at inducing the apoptotic effects on SP WPE1-NB26 cells (Figure 9). This indicates that these drugs can sensibilize the immature and chemoresistant PC cells to the cytotoxic effects of docetaxel.

## Discussion

There has been significant improvement in the diagnosis of early stage PCs by using a combination of screening tests including digital rectal examination, the measurement of serum prostate-specific antigen (PSA) in blood, histological analyses of the expression levels of PC-specific biomarkers in transrectal ultrasound-guided needle biopsy of prostatic tissues and imaging and genetic tests [6], [7], [62]–[65]. These screening tests have led to the identification of previously undetected cases of PCs. In spite of this important advancement, there remains a lack of accurate sensibility and specificity of the current diagnostic and prognostic biomarkers, including the PSA [6], [7], [63]–[66]. Combined with the limited tissue samples available in core biopsy specimens and heterogeneity of the disease, these factors underline the urgent need to validate novel PC-specific biomarkers to optimize the current detection tests and allow individualized treatment of PC patients [3], [6], [7], [63]–[67]. More recently, the development of different multiplex strategies by using new biomarkers such as α-methyl CoA-racemase “AMACR”, pAkt and p63 with the conventional histological markers, including high molecular weight cytokeratin “34betaE12”, has given promising results for optimizing conventional screening tests and improving the accuracy of diagnosis and prognosis of PC patients [68]–[71]. Data from immunhistochemical analyses in the present investigation have indicated that EGFR, pAkt, NF-κB p65 and MIC-1 were overexpressed in the same subset of localized PC tissue specimens as compared to adjacent and normal prostatic tissues during PC progression to locally advanced disease as well as in bone metastasis specimens from PC patients (Figures 1, 2, 3; Table 1). Moreover, the immunofluorescence analyses of these distinct molecular biomarkers with the CD133 stem cell-like marker have also indicated that these oncogenic products were overexpressed in a small subset of CD133^+^ PC cells and the bulk mass of CD133^−^ PC cells in prostatic adenocarcinoma specimens relative to normal prostatic tissues (Figure 4 and Figure S1). These data suggest the potential implication of these oncogenic products in the malignant transformation of PC stem/progenitor cells with stem cell-like features and their progenies in clinical settings.

Accordingly to these results, prior studies have also indicated that a progressive increase of the expression and/or activation of the individual biomarker EGFR, pAkt, NF-κB p65 or MIC-1 often occurred in PC stem/progenitor cells and their progenies during the PC progression to AI, invasive and metastatic disease states [15], [16], [18]–[21], [23], [25]–[27], [30]–[39],[52]. The enhanced expression of these oncogenic products was directly associated with treatment resistance, disease recurrence and a poor outcome and survival of PC patients [15], [16], [18]–[21], [23], [25]–[27], [30]–[39], [52]. More specifically, the characterization of PC cells and animal models relevant to prostate carcinogenesis has indicated that the persistent activation of EGFR and PI3K/Akt in PC cells with stem cell-like features may contribute to their high self-renewal and tumorigenic capacities, treatment resistance and tumor re-growth [13]–[15], [21], [58], [72]–[74]. It has also been shown that the enhanced expression and constitutive activation of NF-κB, which may be induced through diverse growth factor cascades, including EGFR/PI3K/Akt and chemotherapeutic drugs such as docetaxel in PC cells, may result in an increase of the expression levels of numerous oncogenic products and anti-apoptotic factors [22], [24]–[26], [30], [75], [76]. The gene products induced *via* the NF-κB transcription factor include MIC-1, interleukin-6, Bcl-2 and survivin that in turn may promote the survival, invasion and chemoresistance of PC cells [22], [24]–[26], [30], [75], [76]. Especially, a marked increase of the MIC-1 level has been observed in PC cells and serum samples during the early stage of prostate carcinogenesis and transition from localized PCs to AI and metastatic disease states [30], [31], [39], [77], [78]. The enhanced expression of MIC-1 was associated with the development of resistance to docetaxel and mitoxantrone and a poor outcome and survival of the patients [30], [31], [39], [77], [78]. Consequently, the combined immunohistochemical analyses of EGFR, pAkt, NF-κB p65 and/or MIC-1 expression levels with the current histological biomarkers in the patient's prostate biopsies could be helpful in predicting the risk of PC progression to locally invasive and metastatic stages, treatment resistance and biochemical disease recurrence. A combination of these molecular biomarkers could also be used to optimize the individualized treatment of PC patients that are susceptible to respond to the cytotoxic agents targeting these oncogenic products.

In addition, the results of the present investigation have also revealed that the targeting EGFR, PI3K/Akt, NF-κB or MIC-1 by using gefitinib, LY294002/Akt inhibitor VIII, partenolide or anti-MIC-1 antibody significantly inhibited the basal and EGF-promoted prostasphere formation by CD133^+^ SP WPE1-NB26 cells endowed with stem cell-like features (Figures 5 and 6). These data suggest that these oncogenic products may contribute to the self-renewal capacity of CD133^+^ SP WPE1-NB26 cells. In particular, the data have indicated that a treatment with exogenous EGF of CD133^+^ SP WPE1-NB26 cells significantly enhanced their prostasphere-forming ability at least in part through the stimulation of PI3K/Akt and NF-κB/MIC-1 suggesting that EGF/EGFR system can provide important functions for the high self-renewal ability of these immature PC cells (Figure 6). Of therapeutic interest, all of tested pharmacological agents, including gefitinib, LY294002/Akt inhibitor VIII, partenolide or anti-MIC-1 antibody, were also effective at reducing the viability of SP and non-SP WPE1-NB26 cell fractions (Figure 7). Importantly, all of these cytotoxic agents also induced the disintegration of CD133^+^ SP WPE1-NB26 cell-derived prostaspheres and apoptotic effects through the caspase activation, and sensibilized these chemoresistant SP cells to apoptotic effects induced by the current chemotherapeutic drug, docetaxel (Figures 8 and 9). Collectively, these results support the clinical relevance to use a combination of these distinct oncogenic products as molecular targets to develop a multitargeted therapy for eradicating the total PC cell mass. Consistent with this, our recent works combined with several prior studies have revealed that the down-regulation of the expression and/or activity of EGFR, PI3K/Akt, NF-κB or MIC-1 signaling element resulted in growth inhibition and a high rate of apoptotic death of androgen-dependent, AI and metastatic PC cell lines, including PC stem/progenitor cells [34], [36], [38], [58], [75], [76], [79], [80]. More specifically, we have shown that the co-targeting of EGFR and sonic hedgehog pathways by using gefitinib and cyclopamine with the chemotherapeutic drug, docetaxel or mitoxantrone resulted in supra-additive anti-proliferative, anti-invasive and apoptotic effects on diverse invasive and metastatic PC cell lines, including CD133^+^ SP WPE1-NB26 cells, as compared to individual agents and two-drug combinations [36], [52], [79]. Moreover, the inhibition of PI3K/Akt/mTOR signaling elements by using a specific inhibitor of PI3K (LY294002), mTOR (rapamycin, RAD-001 (40-*O*-(2-hydroxyethyl)-rapamycin or CCl-779) or dual PI3K/mTOR inhibitor (PI-103 or NVP-BEZ235) has also been observed to induce a growth inhibition and cytotoxic effects on the CD133^+^/CD44^+^ cell fraction and the bulk PC cell mass detected by cytometric analyses [74]. It has also been reported that a treatment with the sesquiterpene lactone, partenolide, of parental and CD44^high^ and CD44^−/low^ PC cell fractions isolated from metastatic cell lines (DU145, PC3, VCAP and LAPC4) and primary PC tumor cells induced cytotoxic effects *in vitro* through an inhibition of NF-κB and Src-related signaling components and inhibited the tumor growth of CD44^high^ DU145 cell xenograft models *in vivo* [80]. Importantly, the natural compound partenolide was also effective at inducing the cytotoxic effects on CD133^+^ primary prostate tumor cells while CD133^+^ normal cells from benign prostate hyperplasia were insensitive to this treatment type *in vitro* [58].

Taken together, these observations suggest that the combined histological analyses of the expression levels of EGFR, pAkt, NF-κB p65 and/or MIC-1 with the current molecular biomarkers used clinically may constitute a promising strategy to optimize the efficacy of diagnosis, prediction of prognosis, long-term follow-up and choice of therapeutic treatment of PC patients. These data also support the therapeutic interest in using the specific inhibitory agents of EGFR, PI3K/pAkt, NF-κB and/or MIC-1 for eradicating PC stem/progenitor cells and their progenies, and thereby improving the anticarcinogenic efficacy of current anti-hormonal and first-line docetaxel-based chemotherapeutic regimens against locally invasive and metastatic CRPCs and preventing the disease relapse and the death of patients.

## Supporting Information

Figure S1 **Immunofluorescence analyses of expression levels of activated Tyr^1173^-pEGFR signaling element and its co-localization with a CD133 stem cell-like marker in non-malignant and malignant prostatic tissues.** The double immunofluorescence analyses of the co-localization of the expression of markers in normal prostate and prostatic adenocarcinoma specimens from patients was simultaneously done with fluorescein-labeled anti-Tyr^1173^-pEGFR (green) plus phycoerythrin-labeled anti-CD133 antibody (red) after blocking with goat serum as described in Materials and Methods. The arrows indicate a double staining (yellow/purple) detected by confocal analyses, which is indicative of the co-localization of these markers. Representative pictures are shown at the original magnification of ×630.
(TIF)
