# Supplementary figures and images for: Pathobiological Implications of the Expression of EGFR, pAkt, NF-κB and MIC-1 in Prostate Cancer Stem Cells and Their Progenies
Source: PLoS One. 2012 Feb 23;7(2):e31919. doi: 10.1371/journal.pone.0031919 (PMC3285632; doi:10.1371/journal.pone.0031919)

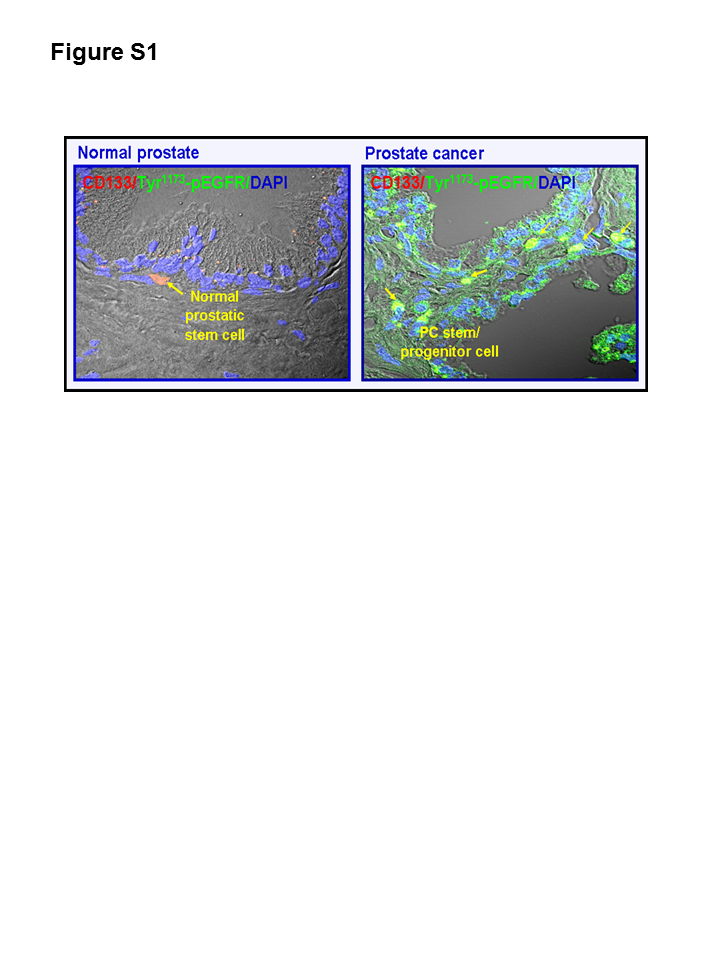

Supplement: Figure S1 — Immunofluorescence analyses of expression levels of activated Tyr1173-pEGFR signaling element and its co-localization with a CD133 stem cell-like marker in non-malignant and malignant prostatic tissues. The double immunofluorescence analyses of the co-localization of the expression of markers in normal prostate and prostatic adenocarcinoma specimens from patients was simultaneously done with fluorescein-labeled anti-Tyr1173-pEGFR (green) plus phycoerythrin-labeled anti-CD133 antibody (red) after blocking with goat serum as described in Materials and Methods. The arrows indicate a double staining (yellow/purple) detected by confocal analyses, which is indicative of the co-localization of these markers. Representative pictures are shown at the original magnification of ×630. (TIF) [file pone.0031919.s001.tif]
